# Metabolic Characterization of Advanced Liver Fibrosis in HCV Patients as Studied by Serum ^1^H-NMR Spectroscopy

**DOI:** 10.1371/journal.pone.0155094

**Published:** 2016-05-09

**Authors:** Nieves Embade, Zoe Mariño, Tammo Diercks, Ainara Cano, Sabela Lens, Diana Cabrera, Miquel Navasa, Juan M. Falcón-Pérez, Joan Caballería, Azucena Castro, Jaume Bosch, José M. Mato, Oscar Millet

**Affiliations:** 1 CIC bioGUNE, Derio, Bizkaia, Spain; 2 Liver Unit, Hospital Clinic, IDIBAPS, University of Barcelona, Barcelona, Spain; 3 ONE WAY LIVER METABOLOMICS SL, Derio, Bizkaia, Spain; 4 Centro de Investigación Biomédica en Red de Enfermedades Hepáticas y Digestivas (CIBERehd), Barcelona, Spain; 5 Ikerbasque, Basque Foundation for Science, Bilbao, Bizkaia, Spain; Inserm, U1052, UMR 5286, FRANCE

## Abstract

Several etiologies result in chronic liver diseases including chronic hepatitis C virus infection (HCV). Despite its high incidence and the severe economic and medical consequences, liver disease is still commonly overlooked due to the lack of efficient non-invasive diagnostic methods. While several techniques have been tested for the detection of fibrosis, the available biomarkers still present severe limitations that preclude their use in clinical diagnostics. Liver diseases have also been the subject of metabolomic analysis. Here, we demonstrate the suitability of ^1^H NMR spectroscopy for characterizing the metabolism of liver fibrosis induced by HCV. Serum samples from HCV patients without fibrosis or with liver cirrhosis were analyzed by NMR spectroscopy and the results were submitted to multivariate and univariate statistical analysis. PLS-DA test was able to discriminate between advanced fibrotic and non-fibrotic patients and several metabolites were found to be up or downregulated in patients with cirrhosis. The suitability of the most significantly regulated metabolites was validated by ROC analysis. Our study reveals that choline, acetoacetate and low-density lipoproteins are the most informative biomarkers for predicting cirrhosis in HCV patients. Our results demonstrate that statistical analysis of ^1^H-NMR spectra is able to distinguish between fibrotic and non-fibrotic patients suffering from HCV, representing a novel diagnostic application for NMR spectroscopy.

## Introduction

The term liver disease gives name to a plethora of pathologies, with more than a hundred forms caused by a variety of factors. Disease onset spans through all stages of life, from infants to older adults. When it becomes chronic, liver disease represents an important and increasing global health problem [1], and despite the rather high incidence and severe consequences, the number of patients suffering from chronic liver disease still remains largely underestimated. Most forms of chronic liver disease are accompanied by liver fibrosis (LF), i.e. which constitutes an excessive accumulation of extracellular matrix proteins that distorts the normal parenchymal structure of the liver [2]. Early diagnosis and classification of the degree of liver fibrosis is, therefore crucial to select the most appropriate therapy, since drugs targeting inflammation and cell injury are more effective at earlier to intermediate stages of the disease.

Among the existing methods to detect liver disease and fibrosis, liver biopsy is considered the gold standard and classifies fibrosis according to different semiquantitative and validated histological scores. The METAVIR system scores fibrosis on a 4 level scale, where F0 indicates the absence of fibrosis, and F4 is a fully developed cirrhosis [3]. Patients with stage F2 or higher are considered to have significant fibrosis. However, liver biopsy is an invasive method that remains an imperfect reference, where accuracy is affected by many factors including sample size [4, 5], or sampling location of the biopsy specimen [6], and the etiology of the disease [7, 8]. Alternatively, several non-invasive techniques can be used to identify LF. Transient elastography is the most popular imaging technique evaluating liver stiffness, but its accuracy in predicting cirrhosis (the terminal stage of fibrosis) strongly depends on the observer's expertise. Therefore, the interest in identifying and staging LF by means of molecular serum markers has increased over the last years. The ideal biomarker for LF should be non-invasive, unbiased by inflammatory processes, highly sensitive and specific to identify early disease stages, active fibrogenesis and advanced fibrosis (cirrhosis). Available serum biomarkers can be divided into indirect markers of liver function and direct markers for extracellular matrix turnover [9, 10]. Unfortunately, these biomarkers are not liver specific, have low sensitivity, and/or still require extensive validation.

One of the most efficient ways to identify new biomarkers is using metabolomics, i.e. the comprehensive metabolite analysis in a biological sample [11]. Liver diseases have been the subject of metabolomic analyses, specifically to investigate the development of hepatic fibrosis and cirrhosis in rat and human samples [12]. Although these reports provide significant advances in the field, the metabolic alterations associated with liver disease are not yet well understood. In fact, there are reported metabolomic studes focusing on the progression of LF in rats [13–16] but not in humans.

Common techniques applied to metabolomics are NMR spectroscopy [17], GC/MS [18] and LC/MS [19, 20]. NMR is an excellent technique for profiling biofluids, and is especially useful for characterizing complex solutions. It is quantifiable, reproducible, non-selective and non-destructive, while not as sensitive as other techniques like GC/MS or LC/MS. NMR based metabolic profiling of biofluids (e.g., serum and urine) has already been widely used to search for potential biomarkers in several diseases [21]. Previously, a pilot study involving NMR spectrometry on urine samples infection demonstrated its potential for the diagnosis of HCV in clinical practice [22]. Many other papers have analyzed different types of liver diseases using also NMR spectroscopy (review by [23]), but no studies have been focus on cirrhosis by HCV infection. The objective of our work is to demonstrate the suitability of ^1^H NMR spectroscopy in characterizing the advanced LF metabolism induced by hepatitis C virus (HCV).

We analyzed serum samples obtained from HCV patients with cirrhosis (the later stage of liver fibrosis) (n = 27) and controls from HCV patients without detectable fibrosis (n = 30) in their liver biopsies. Our results show that statistical analysis of serum ^1^H-NMR spectra can effectively distinguish between these two groups of HCV patients, providing a novel application of NMR spectroscopy.

## Materials and Methods

### Subjects and sample collection

All participants in the study gave informed consent to clinical investigations, according to the Declaration of Helsinki principles and provided written informed consent using an approved consent form. All data were anonymized to protect the confidentiality of individual participants. The study was approved by the Hospital Clínic of Barcelona's Clinical Research Ethics Committee (CEIC).The study initially included a group of 57 well-annotated HCV patients with chronic hepatic disease from the Hospital Clínic de Barcelona. The pool of patients for the current study included two classes: group 4 (G4) comprises 27 patients with chronic HCV liver disease and stage F4 fibrosis, while control group 0 (G0) comprises 30 HCV patients without fibrosis (stage F0), all of them diagnosed by liver biopsy. Blood was drawn from patients presenting to clinic after overnight fast. Whole blood samples were immediately processed for serum and samples were stored at −80°C until retrieval. Blood samples were also used to measure routine laboratory markers and biomarkers, and demographic characteristics were obtained at the same time.

All patients were followed up in the out-patients visits at Hospital Clinic. No patient was receiving antiviral therapy at the moment of the blood extraction. Most of the patients with F0 fibrosis did not show any symptoms and did not receive concomitant or relevant medication. On the other hand, patients with F4 fibrosis (compensated cirrhosis) were more prone to have medical prescriptions. The most common therapies in cirrhotic patients were diuretics, beta-blockers, or pain-killers. At the moment of serum extraction, all of them were under a free diet without any specific restriction related with the liver disease. No patient consumed significant alcohol. In cirrhotic patients (F4) the prevalence of hyperglycemia’s disorders may be as high as 30%; however, most of them may be controlled with glucose nutritional restrictions without therapeutic intervention. Five patients were eventually excluded from the analysis for the reasons described in the Results section.

### NMR spectroscopy

For NMR analysis, serum samples were left to thaw, and aliquots of 100 μL were mixed with 200 μL phosphate buffer (pH 7.0) containing 5 mmol/L TSP (Trimethylsilyl propionate) and 5% v/v D_2_O. The final mixtures were transferred to 3-mm NMR tubes. The pure metabolite molecules used for referencing were all obtained from Sigma-Aldrich (St. Louis, MO, USA).

All ^1^H-NMR spectra were measured at 300 K on a Bruker Avance III 600 MHz spectrometer (Bruker Biospin, Germany). Three complementary ^1^H NMR spectra were recorded per sample. A standard ^1^H spectrum with water suppression (using a binomial 3-9-19 pulse) and 1.5 Hz/point resolution was acquired within 100 seconds (using 32 transients and a total interscan recovery delay of 2.2 s). The same experiment was then repeated (64 transients) with an appended 40 ms T_2_ relaxation filter implemented as a CPMG module. Finally, a two dimensional ^1^H,^1^H Total Correlation Spectrum (TOCSY) was recorded within 1 hour (8 transients, 1024 × 192 complex data points, 1.1 s total interscan delay) employing 60ms FLOPSY8 mixing. All spectra were acquired and processed within TopSpin 3.2.6 applying a squared cosine window function, simple zero filling, and automatic zero-order phase correction (manually corrected for the TOCSY spectrum); the spectra were manually referenced against internal TSP (δ = 0.00 ppm). All NMR spectra were also peak-aligned manually using in-house scripts to minimize variation due to peak shift [24].

Only the T_2_ filtered one-dimensional ^1^H NMR spectra were used for automatic statistical analysis owing to their superior clarity, while their standard (not T_2_ filtered) analogues suffered from massive overlap from broad serum protein background signals. The two-dimensional TOCSY spectra were used to identify and assign specific metabolites. The not T_2_ filtered was proven useful to identify broad signals from triglycerides, proteins, cholesterols and phospholipids among others.

### Multivariate statistical analysis

For all statistical analyses the T_2_ filtered ^1^H NMR spectra of the 57 HCV patients (30 non-fibrotic and 27 fibrotic) were subdivided into spectral regions (bins) of 0.03 ppm width, and the pertaining regional integrals (bin intensities) were obtained using the Average Sum method (i. e. to divide the sum by the number of points on the bin). This binning process was performed automatically using the MestReNova software (version 10.0.1, MestreLab Research), and covered the entire spectrum from 0 to 10 ppm. The region 4.5 to 5.15 ppm could not be analysed due to the applied water suppression, while the spectrum was divided into segments ranging from 9.5 to 0.5 ppm. Thus, a total of 262 bin intensities were obtained and analyzed using the metabolomic data processing server MetaboAnalyst 2.0 (http://www.metaboanalyst.ca) [25, 26]. Specifically, data were pre-processed for normalization and scaling, to remove possible bias from sample variability and preparation. Data were normalized to the total spectral area and subsequently scaled (mean-centered and divided by standard deviation of each variable). The normalized features were subsequently analyzed by PCA to detect intrinsic clusters and outliers within the data set. To maximize separation between samples, partial least-squares discriminant analysis (PLS-DA) was applied. Models were tested by 10-fold cross validation using R^2^ and Q^2^ parameters, where R^2^ provides a measure for how much variation is represented by the model and Q^2^ for the goodness of prediction. Permutation test was performed to check over fitting of the PLS-DA models. After building the PLS-DA model, variable importance in projection (VIP) score of each variable was used to rank the identified distinctive features based on their significance in discriminating between control and fibrotic samples. Variables with VIP score>1 were selected as significant bins.

Moreover, features identified as significant in differentiating between F0 and F4 grades were plotted in a receiver operating characteristic (ROC) curve to visualize the predictive ability of these features at different sensitivity and specificity levels using the ROCCET server (http://www.roccet.ca) [27]. ROC curves were generated by Monte Carlo Cross Validation (MCCV) using balanced subsampling. In each iterative MCCV step, two thirds of the samples were used to evaluate the feature importance. The area under the curve (AUC) represents the discriminatory ability of this metabolite as a potential biomarker, with values close to 1 implying a better classification.

The most important distinctive features identified from the PLS-DA VIP model and showing the highest individual ROC values, were subsequently combined linearly into a unique multiparametric predictive model using in-house MATLAB scripts. The risk score (H) of this model was based on the following equation:

$$H=\sum_{i=1}^{n}\beta i\cdot \text{Vi}$$ (1)

where β_i_ is the estimated beta coefficient for variable V_i_ and n accounts for the total number of variables considered. The ability of the obtained multiparametric models to discriminate between HCV patients with and without significant fibrosis was assessed by ROC curve.

### Univariate analysis

Univariate statistics were performed on the selected variables as an alternative measure of variable importance. To determine if changes in bin intensities were statistically significant fold changes, Pearson’s r correlation and non-parametric Wilcoxon rank-sum scores were calculated using MetaboAnalyst 2.0. Altered features were considered significant when pertaining *p*-values were less than 0.05. Finally, a heatmap of the samples was generated to visualize the clusters identified by PLS-DA VIP scores.

### Metabolite identification

The metabolites highlighted as significant hits by PLS-DA (with VIP > 1) were finally identified mainly from the well resolved TOCSY spectra, by comparing their spin systems and chemical shifts with those reported in the literature or deposited in the Madison Metabolomics Consortium Database (http://mmcd.nmrfam.wisc.edu/) and Human Metabolome Database (HMDB) (http://www.hmdb.ca/metabolites/) [28, 29]. Using the standard ^1^H spectrum we were able to identify broad signals from cholesterols or phospholipids. The identity of several metabolites was furthermore confirmed by NMR after addition of the corresponding pure compounds to a mixture of healthy human serum samples.

## Results

### ^1^H-NMR spectra and multivariate analysis

We have verified the applicability of ^1^H NMR spectroscopy in the diagnosis, staging and prognosis of HCV-induced liver disease by analyzing serum samples of 57 HCV patients in the non-fibrotic (G0, 30 patients) and cirrhotic (G4, 27 patients) stages. For statistical analysis of the T_2_-filtered one-dimensional ^1^H spectra we extracted and compared the signal intensity of the spectral regions (bins) of 0.03 ppm width. To examine whether any age-or sex related metabolic differences existed in our subject groups, PCA of ^1^H-NMR spectra between control and patient subjects was performed. No age- or sex-related groupings were observed in the PCA score plots, indicating that the majority of variance in the data is not related to any possible metabolic differences in the age or gender of the subject (S1 Fig).

An initial exploratory PCA of the bin intensities produced a first survey of the metabonomic dataset and unbiased data clustering and revealed 5 strong outliers, of which 4 belonged to group G0 and one to G4 (data not shown). The fact we did not get enough metadata from one of the samples to compare with all other, coupled with this sample was located out from the Hotelling T^2^ distribution ellipses, led us to exclude it from the study. Other two outliers presented hemolysis, and the other two produced NMR spectra of poor quality and water suppression. These five outliers were excluded from further analysis. PCA also revealed one weak outlier in the Hotelling T^2^ distribution ellipses (drawn at T^2^ = 0.95, S2A Fig) that was nevertheless included in the study. The remaining set of 51 patients showed well-distributed demographic, clinical and biological parameters (listed in metadata Table 1), and was subjected to further analysis. A scatter plot of PC1 vs. PC2 scores indicated no unsupervised separation trends between fibrosis patients in stage F0 and F4 (S2A Fig) while the pertaining groups G0 and G4 can be partially discriminated when including the third principal component, PC3 (S2B Fig). The difficulty in separating both groups likely reflects the complexity of the serum sample and multifactorial variability.

**Table 1:** Clinical Metadata of the Studied Patient Population.

	METAVIR stage	
Metadata	F0 (n = 26)	F4 (n = 26)
Age (years)^*a*^	40.63± 16.35	58.15± 9.03
Male gender^*b*^	46.15	61.54
Genotype VCH ^*b*^		
1a	7.69	16.00
1b	61.54	64.00
2	3.85	4.00
3	11.54	8.00
4	15.38	8.00
AST (IU/l)^*a*^	39.31± 21.67	120.62± 67.17
ALT (IU/l)^*a*^	65.04± 32.98	140.65± 101.46
GGT (IU/l)^*a*^	32.65± 26.77	66.42± 35.29
ALP (IU/L)^*a*^	159.00± 80.12	247.67± 115.48
Bilirubin (mg/dl)^*a*^	0.61± 0.30	1.07± 0.51
VCHCV (log)^*a*^,^*c*^	5.82± 0.87	6.08± 0.59
Glucose (mg/dl)^*a*^	82.31± 23.21	85.61± 40.35
Platelet (G/L)^*a*^	173.08± 99.26	117.65± 80.97
Cholesterol (mg/dl)^*a*^	143.81± 77.95	122.73± 74.94
BMI (Kg/m^2^)^*a*^	24.08± 9.95	25.14± 7.19

^*a*^ Values are expressed as the average ± standard deviation.

^*b*^ in percent of patients.

^*c*^ Viral load of Hepatitis C virus.

Abbreviations: ALT, alanine aminotransferase; AST, aspartate aminotransferase; ALP, alkaline phosphatase; GGT, γ-glutamyl transferase; BMI, body mass index.

**Fig 1:**
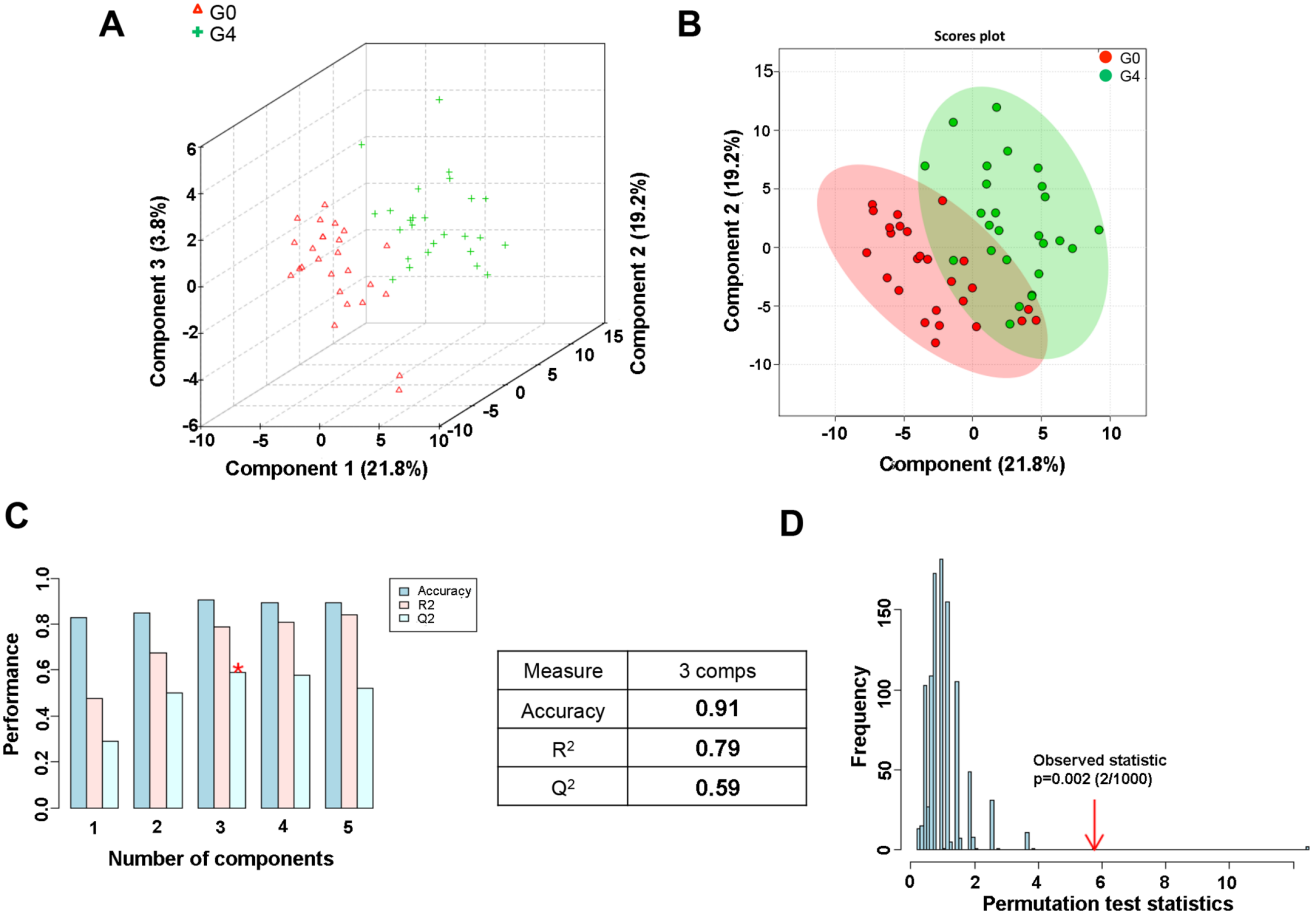
PLS-DA analysis for G0 versus G4 serum samples. (A) Three-dimensional PLS-DA score plot. Red triangles: G0 samples (fibrosis stage F0). Green crosses: G4 samples (fibrosis stage F4). (B) Two-dimensional PLS-DA score plot. Red circles: G0 samples. Green circles: G4 samples. (C) PLS-DA classification using different numbers of components. The red asterisk indicates the best classifier. The inset table summarizes Q^2^, R^2^ and accuracy of the best model. Comps means number of components. (D) Permutation test statistics for 1000 permutations with observed statistic at p < 0.01.

Subsequent PLS-DA corroborated the group separation, where both fibrosis classes are well discriminated by three principal components (Fig 1A), while some overlap persists when considering only PC1 and PC2 (Fig 1B). To evaluate the statistical robustness of the analysis, 10-fold cross-validation was performed and Q^2^ and R^2^ values were deduced. Q^2^ provides an estimate of the predictive power of the PLS-DA models, with a minimal threshold for good separation given by *Q*^2^ > 0.5. Increased R^2^ values (0 < R^2^ < 1) likewise reflect predictive power and variance. Our model scored high R^2^ ≈ 0.8 and Q^2^ ≈ 0.6, confirming good predictive power (Fig 1C). Permutation tests (1000 repeats) yielded a very low *p* ≤ 0.01, indicating that none of the distributions formed by the permuted data is better than the observed statistic based on the original data (Fig 1D). S3 Fig shows the loading plot component 1 *vs*. component 2 resulting after applying PLS-DA to NMR binned spectra of serum of controls and cirrhotic patients.

**Fig 2:**
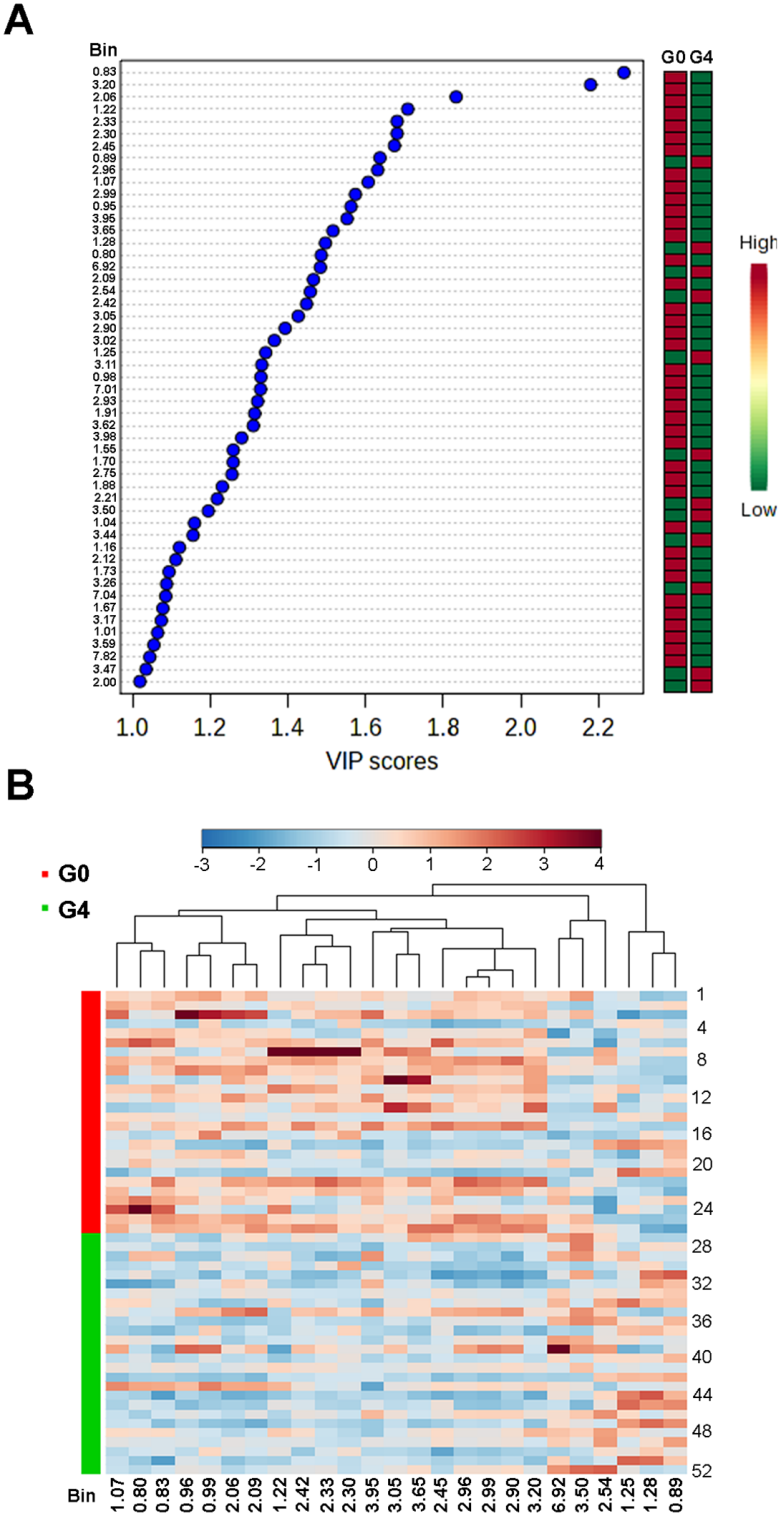
Significant features (binned signals) discriminating between G0 and G4 serum samples. (A) Important features identified by PLS-DA and VIP scores. The colored boxes on the right indicate relative bin integrals for G0 and G4 samples. Variable Importance in Projection is a weighted sum of squares of the PLS loadings taking into account the amount of explained Y-variation in each dimension. (B) Heatmap of unsupervised hierarchical clustering (distance measure using Pearson and clustering algorithm using Ward). The heatmap was constructed from the most significantly differing bins (features), as identified by PLS-DA and VIP scores. Only the top 25 features are shown. Each colored cell on the map corresponds to a relative concentration value, with samples in rows (S2 Table indicates the original name of every sample) and features/compounds in columns. Red and blue colors denote increased and decreased bin integrals, respectively.

Fig 2A and S1 Table show the key differentiating features (i.e., diagnostic binned NMR signals) identified by PLS-DA analysis and sorted by increasing VIP score (only those with a significant VIP value > 1 are included). Taking a VIP cut-off at 1.4, 27 features were found to be significant discriminators between fibrosis classes G0 and G4, while only three features (i.e., the NMR signals at 0.83, 3.20 and 2.06 ppm) showed a VIP > 1.8 for the same discrimination. A heatmap of relative intensity changes of potentially differentiating NMR signals (from the PLS-DA / VIP analysis) was then composed to visualize possible clustering (Fig 2B). Indeed, a clear pattern emerges that shows depletion for the majority of metabolite NMR signals in patients with fibrosis at stage F4 as compared to F0, while a minority of signals increase.

### Univariate analysis

After validating our hypothesis that fibrosis stages F0 and F4 from HCV can be distinguished based on T_2_ filtered one dimensional ^1^H NMR spectra, a univariate analysis was performed to confirm the statistical significance of the distinctive peak regions (bins). A Wilcoxon Mann Whitney test classified 51 bins as statistically significant (p < 0.05; S3 Table) and a correlation analysis (S4 Fig) revealed clusters of bins with a specific pattern of changes that could be quantified in relative terms: positive and negative correlation coefficients indicate metabolites with increased concentration in the G4 and G0 patient groups, respectively. The correlation coefficients are close to ± 0.5, indicating a moderate but significant correlation. S4 Table summarizes the results of the correlation analysis, including the *p*-values from a *t*-test on the correlation coefficients and the false discovery rates (FDR). Both Wilcoxon Mann Whitney test and correlation analysis identified a common set of variable bins between groups G0 and G4, with the bins at 0.83 and 3.20 ppm showing the most significant *p*-values. This result is consistent with that from multivariate analysis, i.e. both methods identified the same significantly changing bins.

### Metabolite identification

**Fig 3:**
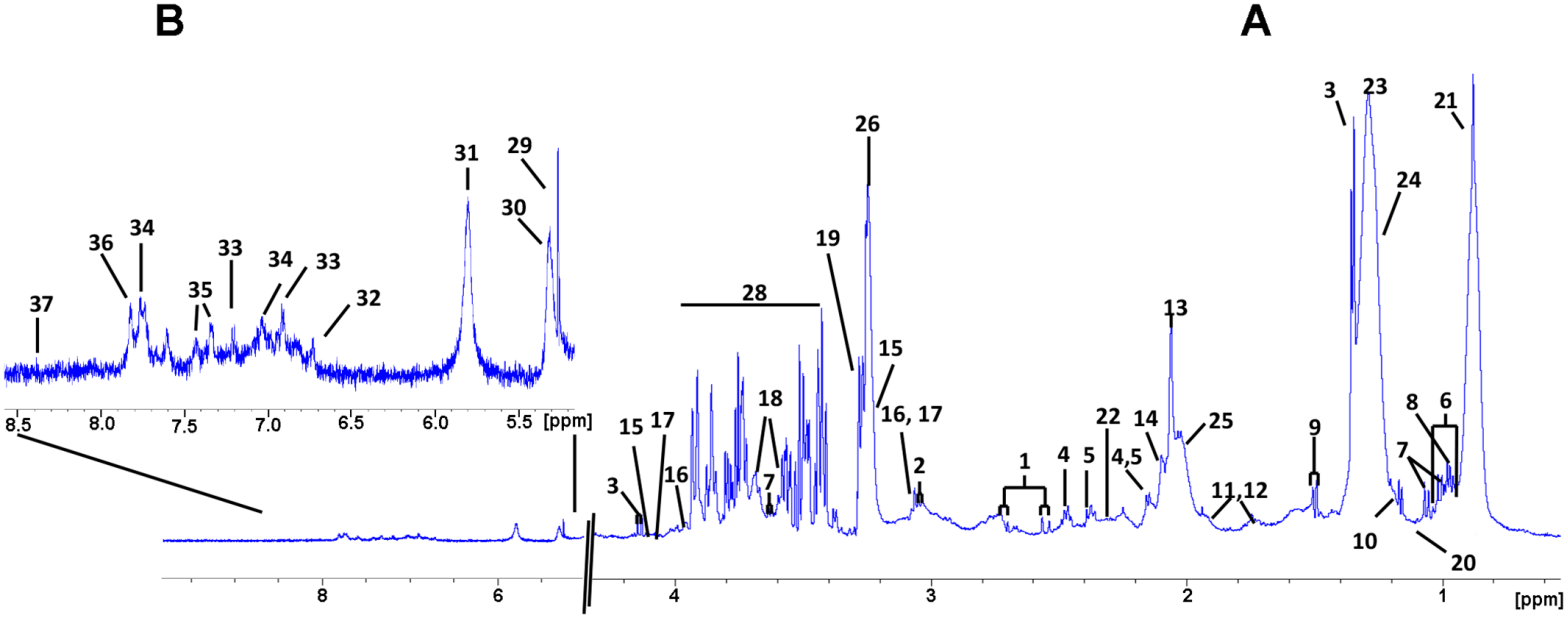
Representative T2-filtered ^1^H- NMR spectrum of human serum sample measured at 300 K, 600 MHz. (A) Full spectrum (0–10 ppm), (B) 35× zoom on the aromatic signal region (5.5–8.5 ppm). Signal assignments were derived by consulting the NMR metabolic profiling database (HMDB), literature references, or from NMR experiments on the pure compounds added to an average serum sample. Spectra were referenced internally against the TSP signal (δ = 0.00 ppm). Assignment numbers correspond to identified metabolites as follows: 1, citric acid; 2, cysteine; 3, lactic acid; 4, glutamine; 5, glutamate; 6, isoleucine; 7, valine; 8, leucine; 9, alanine; 10, 3-hydroxybutyrate; 11, lysine; 12, arginine; 13, Nac1 (N-acetyl of glycoproteins); 14, Nac2; 15, choline; 16, creatine; 17, creatinine, 18, glycerol; 19, TMAO (trimethylamine N-oxide); 20, isobutyric acid; 21, VLDL1 (very low density lipoproteins); 22, acetoacetate; 23, VLDL2; 24, LDL2 (low density lipoproteins); 25, Lipid; 26, GPC (glycerophosphocholine); 27, glucose β-H2; 28, glucose/sugars; 29, α-glucose; 30, lipids; 31, urea; 32, fumaric acid; 33, tyrosine; 34, histidine; 35, phenylalanine; 36, hippuric acid; 37, formic acid.

**Fig 4:**
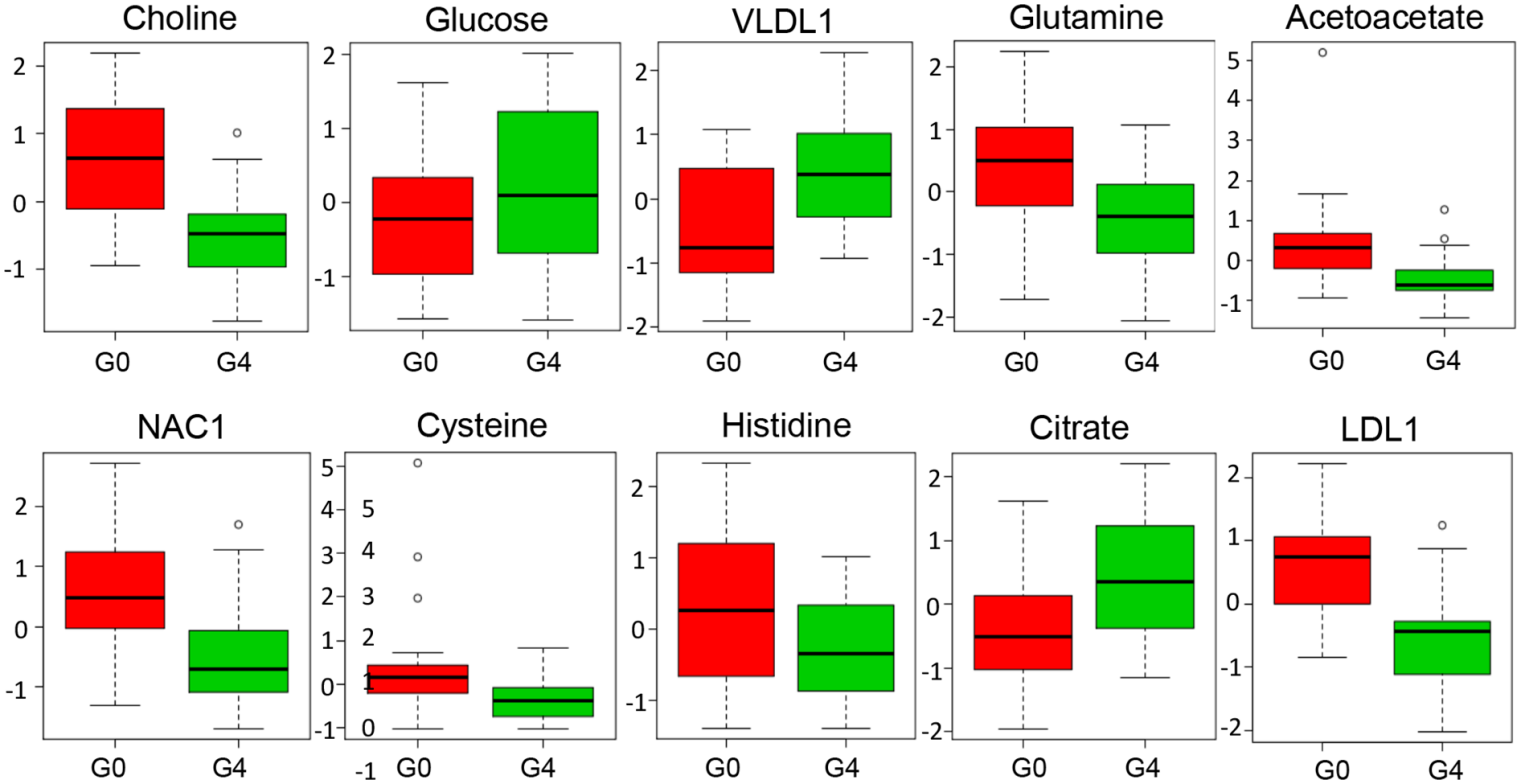
Boxplot of relative concentrations for some significantly altered metabolites (p < 0.05) in serum of G4 (green) and G0 (red) patients. Y axes are represented as relative units. Data were normalized to the total spectral area. Due to this normalization process we obtained negative scale in the Y-axis in some of the bins (Metaboanalyst program analysis). The bar plots show the normalized values (mean +/- one standard deviation). The boxes range from the 25% and the 75% percentiles; the 5% and 95% percentiles are indicated as error bars; single data points are indicated by circles. Medians are indicated by horizontal lines within each box.

For a biochemical interpretation of the observed spectral variations we attempted to assign the bins to their respective metabolites. To that end, each bin was represented by its peak chemical shift value that was then related to a metabolite based on literature. These hypothetical assignments were subsequently confirmed by analyzing spin systems in the serum ^1^H–^1^H TOCSY 2D NMR spectra (S5 Fig), or by recording spectra of the supposed pure compounds added to healthy human serum (S5 Table). Several metabolites with unequivocal assignment are shown in Fig 3. Almost one third of the bins (NMR signal areas) were assigned to different metabolites that include amino acids, organic acids, creatine, creatinine and choline, among others. Yet, a significant number of NMR signal regions could not be unequivocally assigned due to signal overlap and/or low sensitivity. Table 2 presents all the metabolites that were significantly correlated with the model and their variations according to group with significant VIP values > 1 and p-values < 0.05. Most of these metabolites were—detected both, by multivariate and univariate analysis. Correlation analysis and *t*-test furthermore indicated phenylalanine, glucose, hydroxybutyrate and histidine as significant features with *p* values < 0.05 that are also included in Table 2. Broad signals from very low density lipids as VLDL1, VLDL2 were upregulated in cirrhotic patients as compared to controls. Interestingly, low density lipoproteins (LDL) and lipoproteins with higher densities (HDL), were found significantly lower in G4 patients. Choline allows discriminating between G4 and G0 groups: patients with cirrhosis significantly show lower choline signal than patients in the G0 group. Creatinine and creatine were found more intense in the G0 group spectra as compared to the G4 group. Ketone bodies, mainly acetoacetate, but also 3-hydroxibutyrate were significantly downregulated in cirrhotic patients. Several amino acids could also be identified in the spectra. All but phenylalanine were found higher in control samples as compared to advanced fibrotic patients. Citric acid and glucose were also present at higher levels in patients belonging to group G4. Finally, acetyl signals from acid glycoproteins (NAC1 and NAC2) show decreased signal intensity in patients with cirrhosis. Same occurred with glycerol. Fig 4 shows some example boxplots of significantly altered metabolites in G4 vs. G0 patient serum, as detected by univariate and/or multivariate analysis.

**Table 2:** Most important metabolites obtained from the PLS-VIP, Wilcoxon Mann Whitney test and ROC analysis.

Metabolite	Observed δ1H	Assignment	AUROC	p.value
LDL1 ↓	0.84 (bs)	CH_3_(CH_2_)_n_	0.83728	2.95E-06
Choline ↓	3.22 (s)	N-(CH_3_)_3_	0.8284	9.74E-06
Acetoacetate ↓	2.30 (s)	CH_3_	0.80769	0.0012499
NAC1 ↓	2.07 (bs)	NHCOCH_3_	0.7929	0.00028694
Isoleucine +Leucine ↓	0.95 (t) 0.98 (t)	γ-CH_3,_ δ-CH_3_	0.77071	0.0024097
Creatinine + Creatine ↓	3.05 (s), 3.06 (s)	CH_3_ CH_3_	0.76479	0.0073431
LDL ↓	1.23 (m)		0.76036	0.00087216
Glutamate ↓	2.37 (m)	γ-CH_2_	0.75592	0.0010727
Glutamine ↓	2.46 (m)	γ-CH_2_	0.7500	0.0012631
VLDL1 ↑	0.89 (bs)	CH_3(_CH_2_)_n_C =	0.74852	0.0014119
HDL ↓	0.80 (bs)	CH_3_(CH_2_)_n_	0.74556	0.0043228
Asparagine ↓	2.90 (dd)	1/2 β-CH_2_	0.74408	0.001473
Valine ↓	3.62 (d)	α-CH	0.7426	0.012212
Lipid (albumin lysyl) ↓	3.01 (bs)	ε-CH_2_	0.74112	0.0022208
VLDL2 ↑	1.25 (bs)	(CH_2_)n	0.73964	0.01002
Unknown ↓	1.09		0.73669	0.0018143
NAC2 ↓	2.10 (bs)	NHCOCH_3_	0.73077	0.0046624
Citrate ↑	2.55 (d)	1/2 γ-CH_2_	0.72929	0.0049306
Lysine ↓	1.92(m)	β-CH_2_	0.72781	0.011943
Creatine ↓	3.95 (s)	CH_2_	0.71893	0.0025988
Lipid ↑	1.28 (m)	(CH_2_)_n_CO	0.71598	0.0038995
Unknown ↑	6.93		0.71598	0.0043897
Valine ↓	1.01 (d)	γ-CH_3_	0.7145	0.011009
Lysine + Arginine ↓	1.9 (m)	β-CH_2_ β-CH_2_	0.7145	0.016763
Cysteine ↓	3.04 (m)	CH-SH	0.7145	0.0087713
Asparagine ↓	2.93 (dd)	1/2 β-CH_2_	0.70858	0.011321
Arginine + Lysine ↓	1.9 (m)	β-CH_2_ β-CH_2_	0.70562	0.018961
Glutamine ↓	2.45 (m)	γ-CH_2_	0.70562	0.0059592
Asparagine ↓	2.91 (dd)	1/2 β-CH_2_	0.70562	0.007396
Glycerol ↓	3.67 (dd)	1/2 CH_2_	0.70562	0.003731
Arginine ↓	1.69 (m)	γ-CH_2_	0.70414	0.041512
3-hydroxybutyrate ↓	1.16 (d)	γ-CH_3_	0.64793	0.034028
Glucose/Sugars ↑	3.5 (dd)	C2H	0.66124	0.023402
Phenylalanine ↑	3.26 (dd)	1/2 β-CH_2_	0.64645	0.03929
Histidine ↓	7.04 (s)	C_4_H-ring	0.64349	0.040614

A *p*-value < 0.05 from *t*-test between G0 and G4 serum samples was considered to be significant. AUROC: Area under the curve obtained from ROC analysis for every metabolite. Only metabolites with AUROC > 0.7 are shown, except for the last four metabolites with an AUROC > 0.6. First column: Arrows ↓ and ↑ indicate decreased or increased metabolite levels in G4 vs. G0 serum samples. Second column: Chemical shift for every metabolite, signal structure: s, singlet; d, doublet; t, triplet; dd, doublet of doublets; bs, broad signal; m, multiplet; NAC, N-acetyl signals from glycoproteins; LDL, low density lipoproteins; VLDL, very low density lipoproteins; HDL, high density lipoproteins.

### ROC analysis

**Fig 5:**
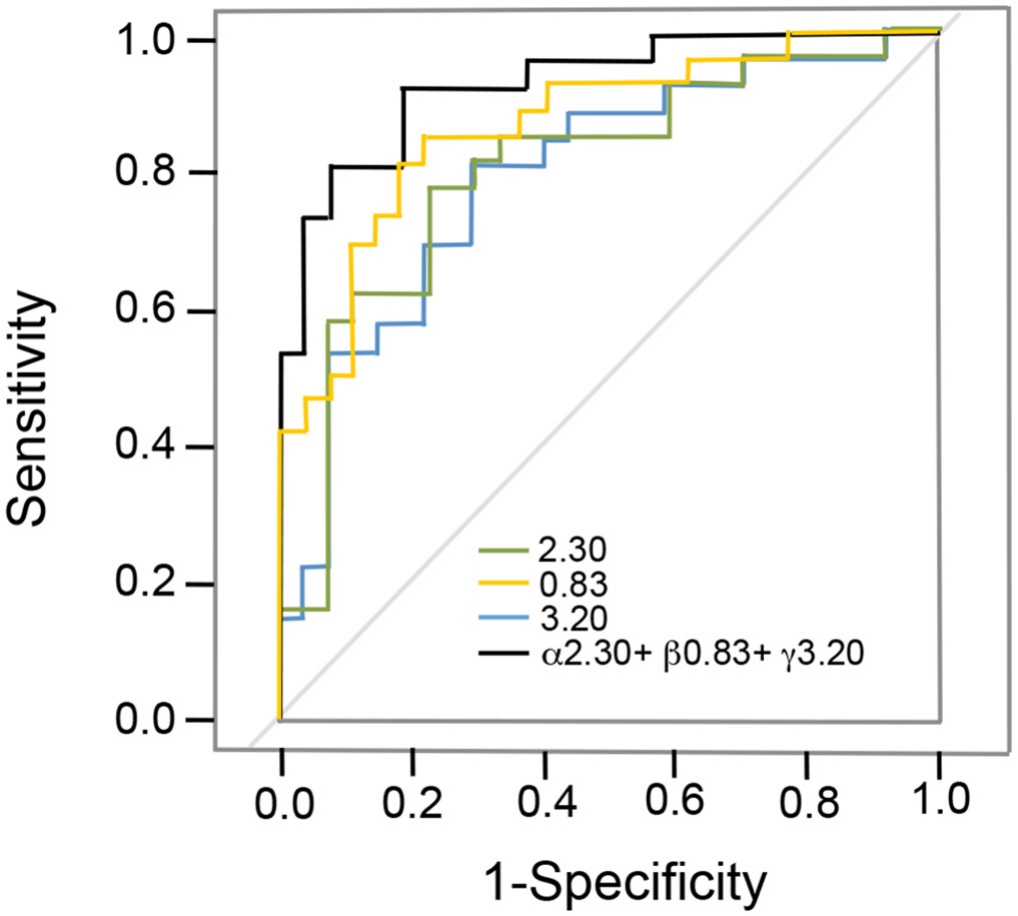
Individual receiver operating characteristic (ROC) curves. The colored curves represent the bins at 0.83 ppm (LDL1), 3.20 ppm (choline) and 2.30 ppm (acetoacetate). The black curve represents our multivariable predictive model described by the linear combination α*2.30 + β*0.83 + γ*3.20 that reaches a cut-off value of -0.316, specificity of 80%, sensitivity of 70%, AUROC score of 0.922, and confidence interval of 95% (0.85 to 0.97).

To better assess the predictive capacity of the PLS-DA classification model, model robustness was assessed using ROC curve analysis. First, ROCCET program was employed to analyze metabolites individually and Fig 5 shows three representative ROC curves. For instance for the bin at 3.20 ppm (corresponding to choline) yields a sensitivity (i.e. percentage of G4 samples correctly classified as positives) and specificity (i.e. percentage of control samples correctly classified as negatives) of 80% and 70%, respectively, with a classification rate of 87% (i.e. total number of correctly classified samples, data not shown). Moreover, the Area Under the pertaining ROC curve (AUROC) was 0.83 (Fig 5), corroborating a high predictive accuracy of the model for this feature. The other two compounds showed also high AUROC values (Fig 5). Table 2 lists AUROC values for the most important metabolites capable of discriminating between G4 and G0 samples. While our results reflect a strong predictive power for some of the assigned metabolites, fibrosis is a complex and multifactorial disease for which, in principle, a multiparametric model based on multiple individual markers should provide a more realistic metabolic description. We therefore developed a simple algorithm to linearly combine different features (selecting metabolites with high AUROC values obtained previously) into a single equation and we subsequently checked for their predictive capacity also running ROC tests, analogous to the ROC value presented above, but reflecting their joint contribution. Thus, by combining the three most significant metabolites identified by multivariate analysis (choline, acetoacetate and LDL1), the AUROC score increased to 0.922 (Fig 5).

## Discussion

Among the etiologies resulting in chronic liver diseases, chronic HCV infection is the leading cause for the development of LF, cirrhosis and hepatocellular carcinoma (HCC). Hence, there is a growing interest in using metabolomics to find biomarkers for this pathology. In the present work we have evaluated whether a metabolomics approach by ^1^H NMR can discriminate between HCV patients with advanced LF (group G4) and those with no signs of LF (group G0). Since metabolomic analyses are very sensitive to a plethora of causative factors we focused on HCV patients with the same etiology and pathophysiology. All patients had a BMI < 26, as an obesity cut-off, an inclusion criteria in the study that intends to minimize the influence of excess adiposity on the generated metabolomic profiles. We found that a distinction between both patient groups is indeed possible by comparing the concentrations of only a reduced set of metabolites, as monitored by ^1^H NMR spectroscopy. Remarkably, the most relevant bins (spectral sections) for distinguishing between groups G4 and G0 emerged from a combination of parametric and non-parametric statistical methods. Moreover, the advantages of using both univariate and multivariate statistics in data mining have recently been highlighted [30], and in the present study, both complementary methods were able to identify virtually the same set of metabolites.

Although our study is preliminary in terms of the number of patients, it is instructive to interpret the most significant metabolites within the context of cellular metabolism. The present data enabled us to draw several conclusions. First, it seems that changes in serum metabolomics profiles reflect activation or impairment in several biologic pathways, mainly energetic metabolism involving glutamine/glutamate, carbohydrates, ketone bodies, and lipids.

Alterations in glucose homeostasis are common in cirrhosis. The liver functions to maintain normal levels of blood sugar by a combination of glycogenesis, glycogenolysis, gluconeogenesis and glycolysis. Our study reveals that glucose is upregulated in the serum of G4 patients with advanced LF as compared to G0 control patients. A similar result was obtained by Amathieu et al [31] when comparing serum from patients with mild or severe chronic liver failure by ^1^H NMR. Alterations in glucose metabolism have been associated with increased severity of liver disease and an elevated risk of liver carcinoma [32]. Compared with the levels in healthy individuals and patients of other liver diseases, autoimmune hepatitis patients exhibited rather high plasma levels of glucose [33]. The increased glucose concentration found in G4 serum could, thus, be due to its reduced metabolization via the tricarboxylic acid cycle, consistent with the insulin resistance commonly found after HCV infection [34, 35]. The upregulation of glucose in serum from G4 patients is also accompanied by a downregulation of glycerol that can be converted to glucose in the liver and provides energy for cellular metabolism. Citrate is likewise upregulated in G4 patient serum suggesting alterations in the metabolic Krebs cycle. Fibrosis grade F4 is considered one step before a potential HCC (F5), and the increased citrate level agrees well with the mitochondrial malfunction observed in liver cancer patient [36]. Cells may use citrate directly to fuel their metabolism and proliferation. Also citrate can be converted to Acetyl-CoA and contribute in the synthesis of fatty acids and cholesterol, essential components of cancer cell membranes [37].

Moreover, sera of G4 patients show lower levels of acetoacetate, the principal component of the ketone bodies produced in the mitochondrial matrix of liver cells in response to carbohydrate deficiency [38]. Gao and colleagues obtained similar results for liver cirrhosis and HCC patients when analyzing their serum by^1^H NMR. Also, 3-hydroxybutirate is downregulated in cirrhotic patients (G4) as compared to G0 samples which has been already observed in hepatitis B virus-infected cirrhosis and alcoholic cirrhosis patients by using ^1^H NMR-based metabonomics [39].

In line with these findings, serum creatine and creatinine levels are also significantly reduced in fibrosis patients. Creatine is synthesized primarily in the liver and is again involved in the general energy supply. The variability in creatinine levels agrees well with the interplay between liver and renal (dis)function in liver disease.

Insulin resistance in liver has been related to oxidative stress, abnormal lipid metabolism and hepatic steatosis after HCV infection [40]. In general, fat accumulation in hepatocytes can be originated from several causes, including a decrease of very-low density lipoprotein secretion. In ^1^H NMR spectra of serum, lipids are detected as broad signals from fatty acid methyl and methylene moieties. Signals at 0.80, 0.84 and 1.23 ppm (from high and low density lipoproteins, HDL and LDL) are downregulated. In several studies, higher HDL levels agree with a good hepatic function while low HDL levels correlate well with chronic liver disease [41]. Chronic HCV is also associated with hypolipidemia [42] and we consistently observe reduced levels (85%) of cholesterol in G4 as compared to G0 patients (Table 1). Contrarily, other lipid concentrations were increased in F4 patients (Table 2), and may be a result of an increased energy requirement for cell proliferation (since cells also use fatty acids to generate their energy).

The liver is the major site of amino acid conversion. Amino acids are needed for synthesis of liver intracellular proteins, plasma proteins, and different compounds, such as glutamine or creatine. The concentrations of amino acids are very often found altered in liver diseases [12, 31, 43, 44]. Amino acid homeostasis is controlled by their appearance and disappearance rates, where the latter comprises conversion into other amino acids, breakdown, excretion and incorporation into proteins. Glutamate and glutamine are glucogenic amino acids involved in glucose regeneration to feed the Krebs cycle. We found out that glutamine and glutamate levels are significantly reduced in G4 patient serum relative to G0 patients. This is consistent with a previous GC-MS study showing that several amino acids branched-chain amino acids (BCAAs), valine, leucine, and isoleucine have been reported to have connections with HCC [45] and were also downregulated in liver cirrhosis patients [46]. In turn, phenylalanine is also upregulated, and patients with fibrosis grade F4 generally suffer an imbalance in the plasma levels of aromatic (AAA) and branched chain amino acids (BCAA). Indeed, a change in the BCAA/AAA ratio has previously been described during hepatic failure [47, 48]. The increase in serum phenylalanine can also be linked to changes in the gut microbiome that catabolizes it, and is well known to be significantly altered in liver disease [49, 50].

We have also observed a decrease in N-acetyl glycoproteins for G4 as compared to G0 patients, which is consistent with the altered carbohydrate content of plasma glycoproteins described for patients of diverse liver diseases [51, 52]. Such variations have also been reported previously for HCC patients, particularly in comparison to cirrhosis patients without cancer [53] or with alcoholic cirrhosis [54]. Overall, our data suggests that a decrease of serum glycoproteins may be associated to fibrosis.

Our results furthermore show increased serum choline concentrations in G4 vs. G0 patients. Wei *et al* were able to distinguish HCC from HCV through metabolite profiling by NMR [55], and choline was found upregulated in HCC patients [56]. Moreover, choline, betaine, and trimethylamine N-oxide (TMAO) seem to be upregulated metabolites in both liver and plasma of rodents after feeding them with diets provoking fatty liver [57]. Bowers *et al*. also found choline to be upregulated in HCC compared to HCV patients, but this was attributed to a bias from the food intake [58]. Our data confirms that choline can be included as a biomarker (see below).

The suitability of the most significantly regulated metabolites as biomarkers was validated by AUROC analysis (Fig 5 and S4 Fig). It reveals that the most informative biomarker for predicting significant fibrosis is choline, with *p* = 9.7e^-06^ (Table 2). Lipids are also very significant markers with a very low *p*-value. Histidine, creatinine and creatine are further metabolites capable of discriminating between fibrosis stages F0 and F4. To enhance their predictive value, we combined the different metabolites into a model with maximal sensitivity, selectivity, and AUROC. Thus, a specific combination of biomarkers is best suited to predict advanced liver fibrosis, and our presented model based on the linear combination of relative concentrations of the three most significantly changing metabolites reaches a sensitivity of 83.5%, specificity of 97.7%, and AUROC score of 0.922 that exceeds the individual AUROC scores for all markers (Fig 5, black curve).

## Conclusions

Liver diseases are an important global health problem, and clinicians are in need for new non-invasive diagnostic biomarkers. In this context, metabolomics emerges as one of the most powerful ways to identify new biomarkers. Here, we have demonstrated the potential of metabolomics by ^1^H NMR spectroscopy on serum samples to discriminate between cirrhotic and non-fibrotic HCV patients. Our results show that ^1^H NMR is a powerful tool to identify and monitor non-invasive biomarkers for advanced liver fibrosis in serum as it is able to directly quantify changes in relative metabolite concentrations that are representative of primary metabolism. A combination of the three most significantly changing metabolites then yields maximal detection sensitivity, specificity and fidelity to distinguish HCV patients in fibrosis stages F0 and F4. Our study paves the way to further NMR analyses on the progression of liver fibrosis (F1 to F3).

## Supporting Information

S1 Fig **Principal component analysis of 1H-NMR spectra corresponding to all the samples included in the study colored according to (A)** age. A: 30-40years, B: 40–50 years, C: 50–60 years and D:> 60 years. **(B)** gender F: Female, M: Male.
(PPTX)

S2 Fig Partial correlation analysis between serum samples from G0 and G4 patients. (A)- 2-D score plot between the selected PCs. Red circles: Control samples (no fibrotic patients or G0). Green circles: Fibrotic samples or G4. (B)- 3-D score plot between selected PCs. Red triangles: samples G0. Green crosses: Fibrotic samples or G4. The explained variances are shown in brackets.
(PPTX)

S3 Fig PLS-DA loadings plot for components 1 and 2. Bins with higher loading are included in S1 Table.
(PPTX)

S4 Fig The 25 most important features selected by correlation analysis. Correlation plot displays a list of features whose relative concentration increased on samples from G0 to G4. The compounds are represented as horizontal bars, with colors in pink indicating positive correlations and that in blue indicating negative correlations. Positive correlation coefficients indicate features upregulated in G4, while negative correlations are associated with lower levels in G0 individuals.
(PPTX)

S5 Fig 2D ^1^H,^1^H-TOCSY spectrum of a representative HCV G0 patient serum sample. Region between 0 and 6 ppm is represented. Assignment of the most significant metabolites is shown.
(PPTX)

S1 Table VIP features. List for the most important features identified by VIP-PLS-DA with VIP > 1. Table shows every bin with the correspondent value for the first 5 components.
(XLSX)

S2 Table List of samples analyzed in this study. Row number correspond to the position of every sample in the heatmap presented in Fig 2B.
(XLSX)

S3 Table Significant features identified by *t*-test. Columns show the different bins, and the corresponding p and False Discovery Rate (FDR) values.
(XLSX)

S4 Table Significant features identified by correlation analysis. Columns show the values of correlation, *t*-test, *p*-value and false discovery rate for every bin.
(XLSX)

S5 Table Identification of metabolites in NMR serum spectra. (A)- Metabolites confirmed to be present in serum. Some of them were identified using ^1^H monodimensional o bidimensional databases. To confirm their presence in human serum, some pure compounds were added to a mix of healthy human serum samples. Chemical shifts that clearly identify those metabolites in our spectra are shown (B)- Metabolites not confirmed to be present in serum using external compounds. Chemical shifts from every metabolite are shown.
(XLSX)

## Abbreviations

NMR: nuclear magnetic resonance
GC/MS: gas chromatography/mass spectrometry
LC/MS: liquid chromatography/mass spectrometry
CPMG: Carr-Purcell-Meiboom-Gill
PLS-DA: partial least squares-discriminated analysis
PCA: principal component analysis
ROC: receiver operating characteristic
