# Supplementary material for: Metabolic Characterization of Advanced Liver Fibrosis in HCV Patients as Studied by Serum 1H-NMR Spectroscopy
Source: PLoS One. 2016 May 9;11(5):e0155094. doi: 10.1371/journal.pone.0155094 (PMC4861296; doi:10.1371/journal.pone.0155094)
Supplement: S1 Fig — Principal component analysis of 1H-NMR spectra corresponding to all the samples included in the study colored according to (A) age. A: 30-40years, B: 40–50 years, C: 50–60 years and D:> 60 years. (B) gender F: Female, M: Male. (PPTX) [file pone.0155094.s001.pptx]

## Slide 1
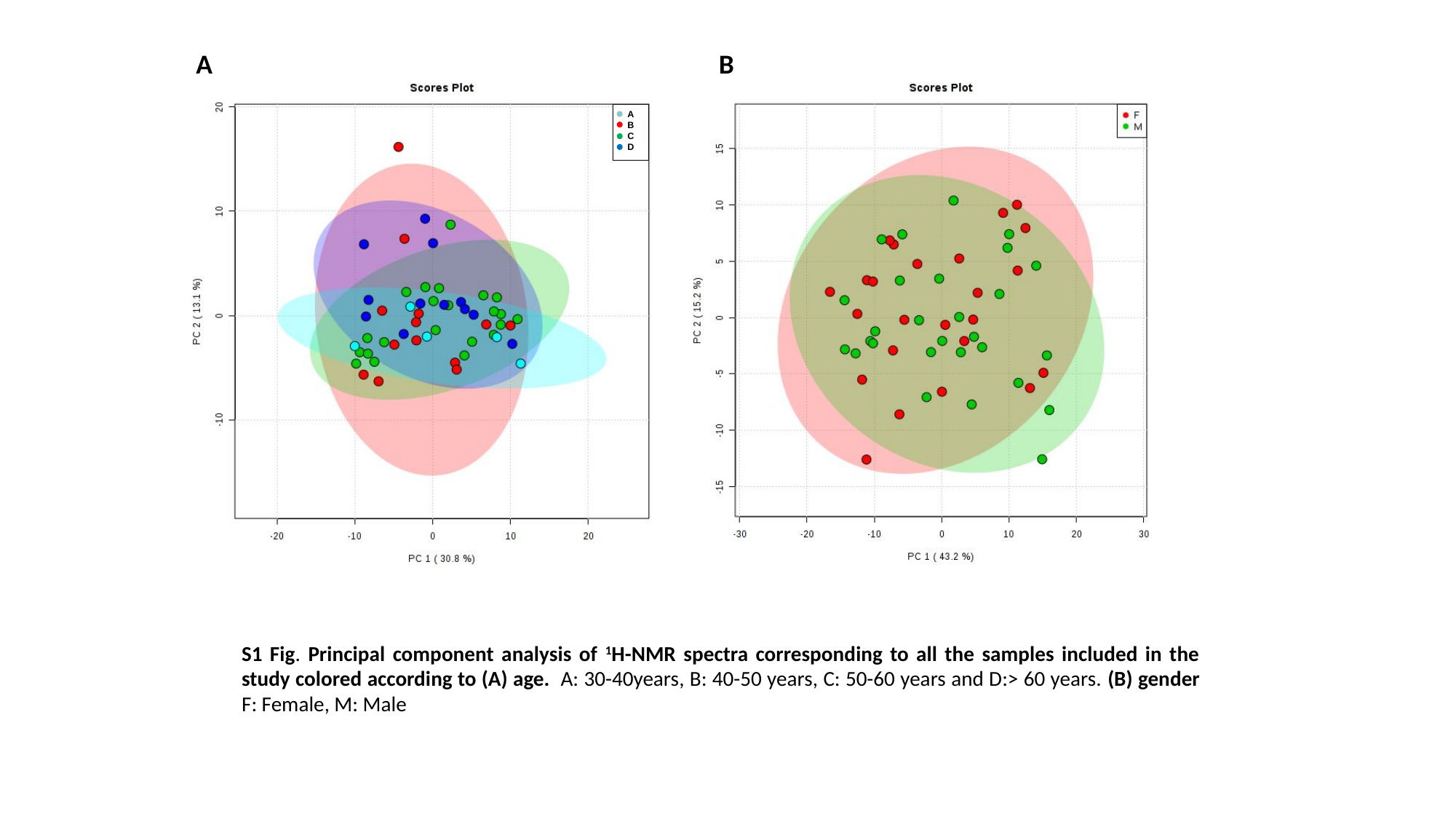

A
B
A
B
C
D
S1 Fig. Principal component analysis of 1H-NMR spectra corresponding to all the samples included in the study colored according to (A) age. A: 30-40years, B: 40-50 years, C: 50-60 years and D:> 60 years. (B) gender F: Female, M: Male
