# Supplementary material for: Metabolic Characterization of Advanced Liver Fibrosis in HCV Patients as Studied by Serum 1H-NMR Spectroscopy
Source: PLoS One. 2016 May 9;11(5):e0155094. doi: 10.1371/journal.pone.0155094 (PMC4861296; doi:10.1371/journal.pone.0155094)
Supplement: S2 Fig — (A)- 2-D score plot between the selected PCs. Red circles: Control samples (no fibrotic patients or G0). Green circles: Fibrotic samples or G4. (B)- 3-D score plot between selected PCs. Red triangles: samples G0. Green crosses: Fibrotic samples or G4. The explained variances are shown in brackets. (PPTX) [file pone.0155094.s002.pptx]

## Slide 1
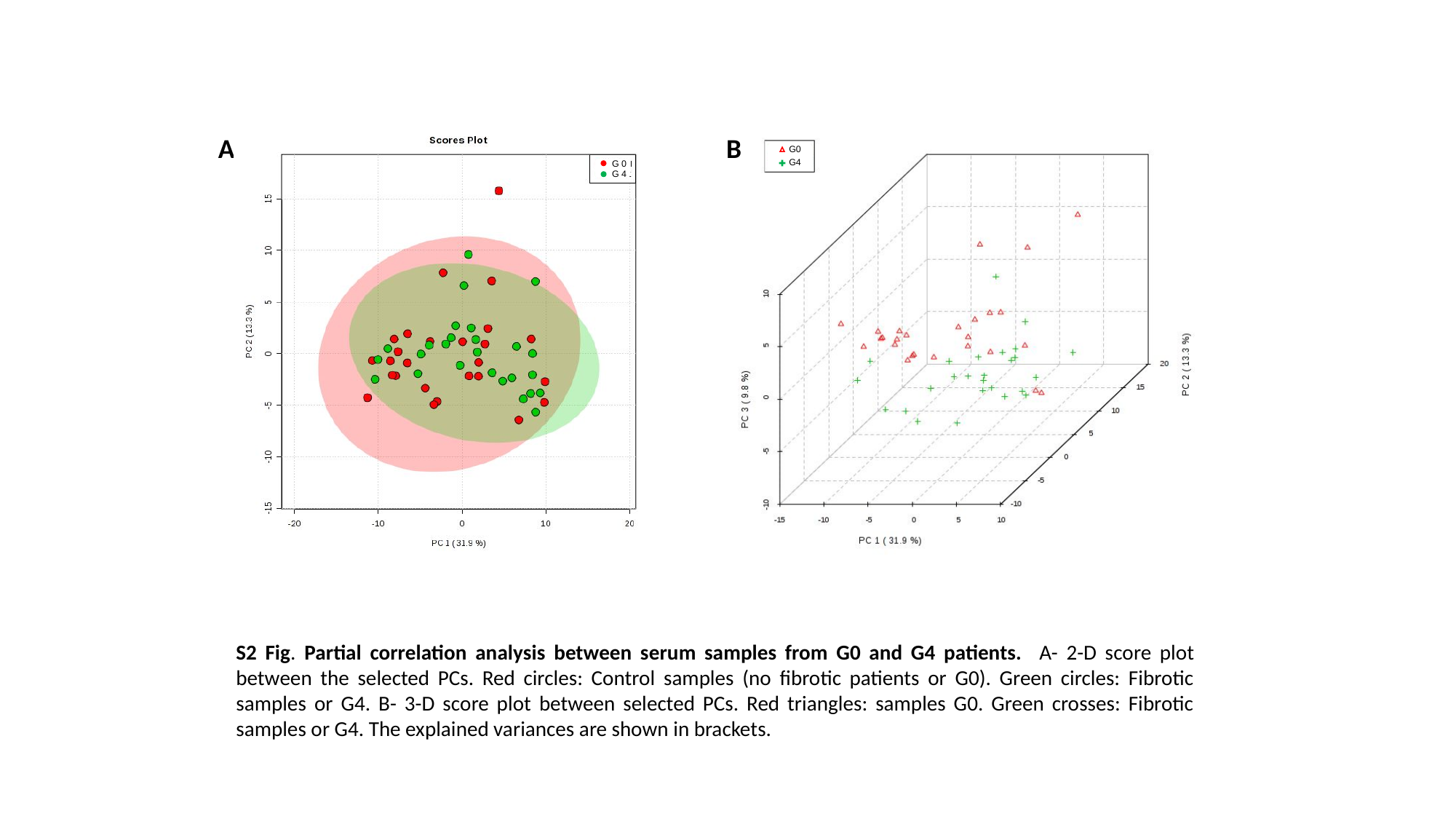

B
A
G0
G4
G 0
G 4
S2 Fig. Partial correlation analysis between serum samples from G0 and G4 patients. A- 2-D score plot between the selected PCs. Red circles: Control samples (no fibrotic patients or G0). Green circles: Fibrotic samples or G4. B- 3-D score plot between selected PCs. Red triangles: samples G0. Green crosses: Fibrotic samples or G4. The explained variances are shown in brackets.
