# Supplementary material for: Metabolic Characterization of Advanced Liver Fibrosis in HCV Patients as Studied by Serum 1H-NMR Spectroscopy
Source: PLoS One. 2016 May 9;11(5):e0155094. doi: 10.1371/journal.pone.0155094 (PMC4861296; doi:10.1371/journal.pone.0155094)
Supplement: S3 Fig — Bins with higher loading are included in S1 Table. (PPTX) [file pone.0155094.s003.pptx]

## Slide 1
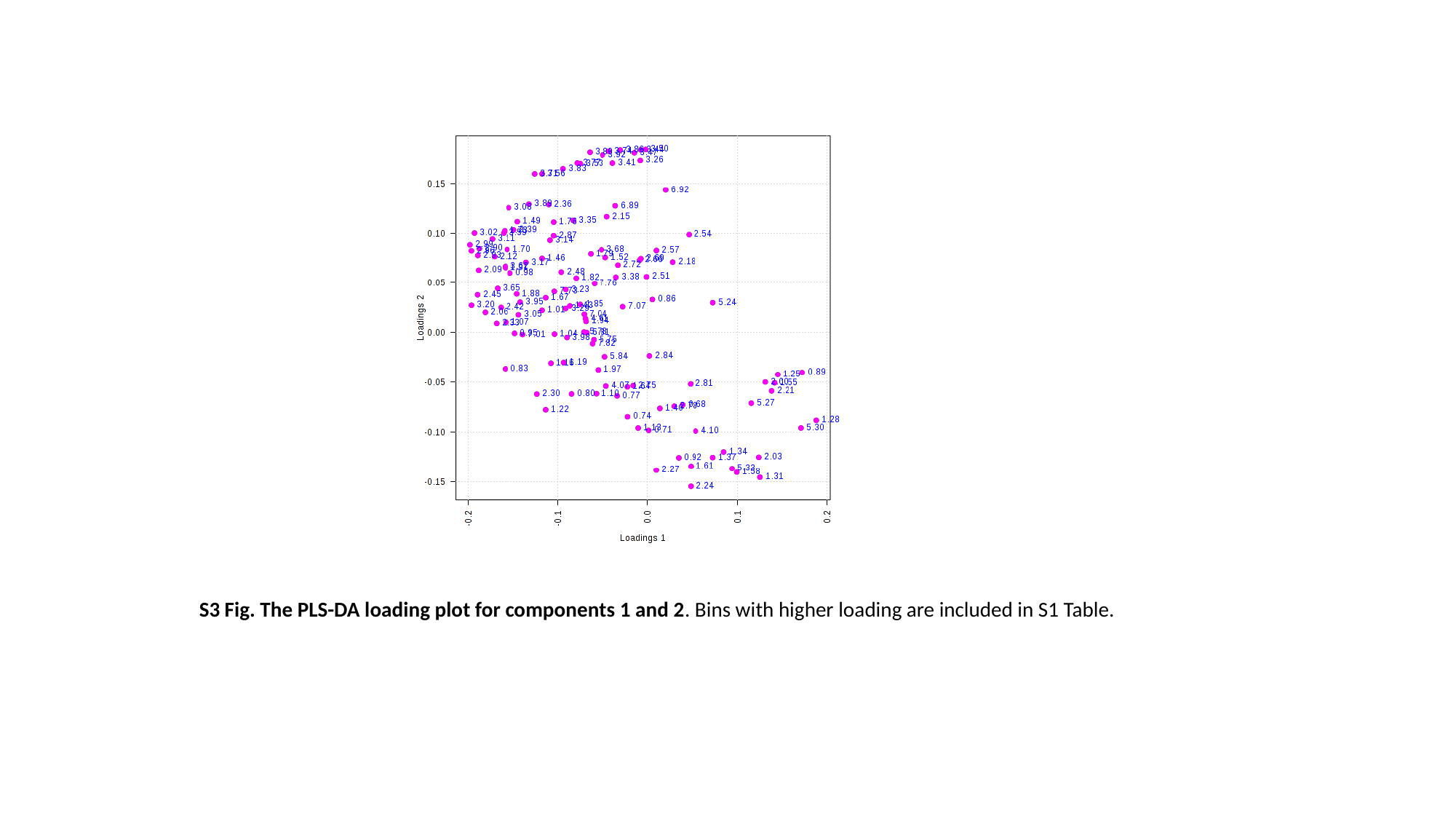

S3 Fig. The PLS-DA loading plot for components 1 and 2. Bins with higher loading are included in S1 Table.
