# Supplementary material for: Metabolic Characterization of Advanced Liver Fibrosis in HCV Patients as Studied by Serum 1H-NMR Spectroscopy
Source: PLoS One. 2016 May 9;11(5):e0155094. doi: 10.1371/journal.pone.0155094 (PMC4861296; doi:10.1371/journal.pone.0155094)
Supplement: S4 Fig — Correlation plot displays a list of features whose relative concentration increased on samples from G0 to G4. The compounds are represented as horizontal bars, with colors in pink indicating positive correlations and that in blue indicating negative correlations. Positive correlation coefficients indicate features upregulated in G4, while negative correlations are associated with lower levels in G0 individuals. (PPTX) [file pone.0155094.s004.pptx]

## Slide 1
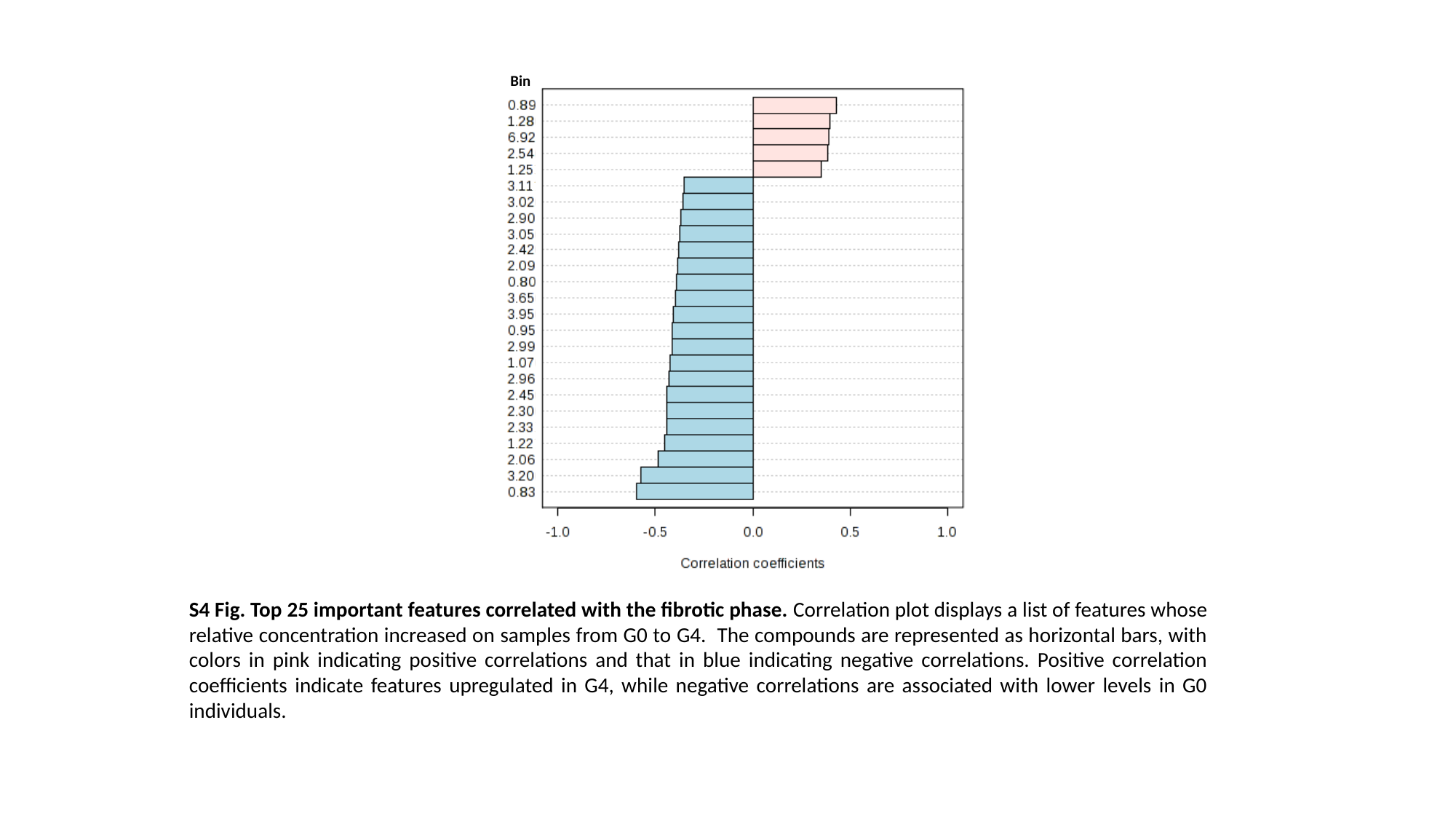

Bin
S4 Fig. Top 25 important features correlated with the fibrotic phase. Correlation plot displays a list of features whose relative concentration increased on samples from G0 to G4. The compounds are represented as horizontal bars, with colors in pink indicating positive correlations and that in blue indicating negative correlations. Positive correlation coefficients indicate features upregulated in G4, while negative correlations are associated with lower levels in G0 individuals.
