# Supplementary material for: Metabolic Characterization of Advanced Liver Fibrosis in HCV Patients as Studied by Serum 1H-NMR Spectroscopy
Source: PLoS One. 2016 May 9;11(5):e0155094. doi: 10.1371/journal.pone.0155094 (PMC4861296; doi:10.1371/journal.pone.0155094)
Supplement: S5 Fig — Region between 0 and 6 ppm is represented. Assignment of the most significant metabolites is shown. (PPTX) [file pone.0155094.s005.pptx]

## Slide 1
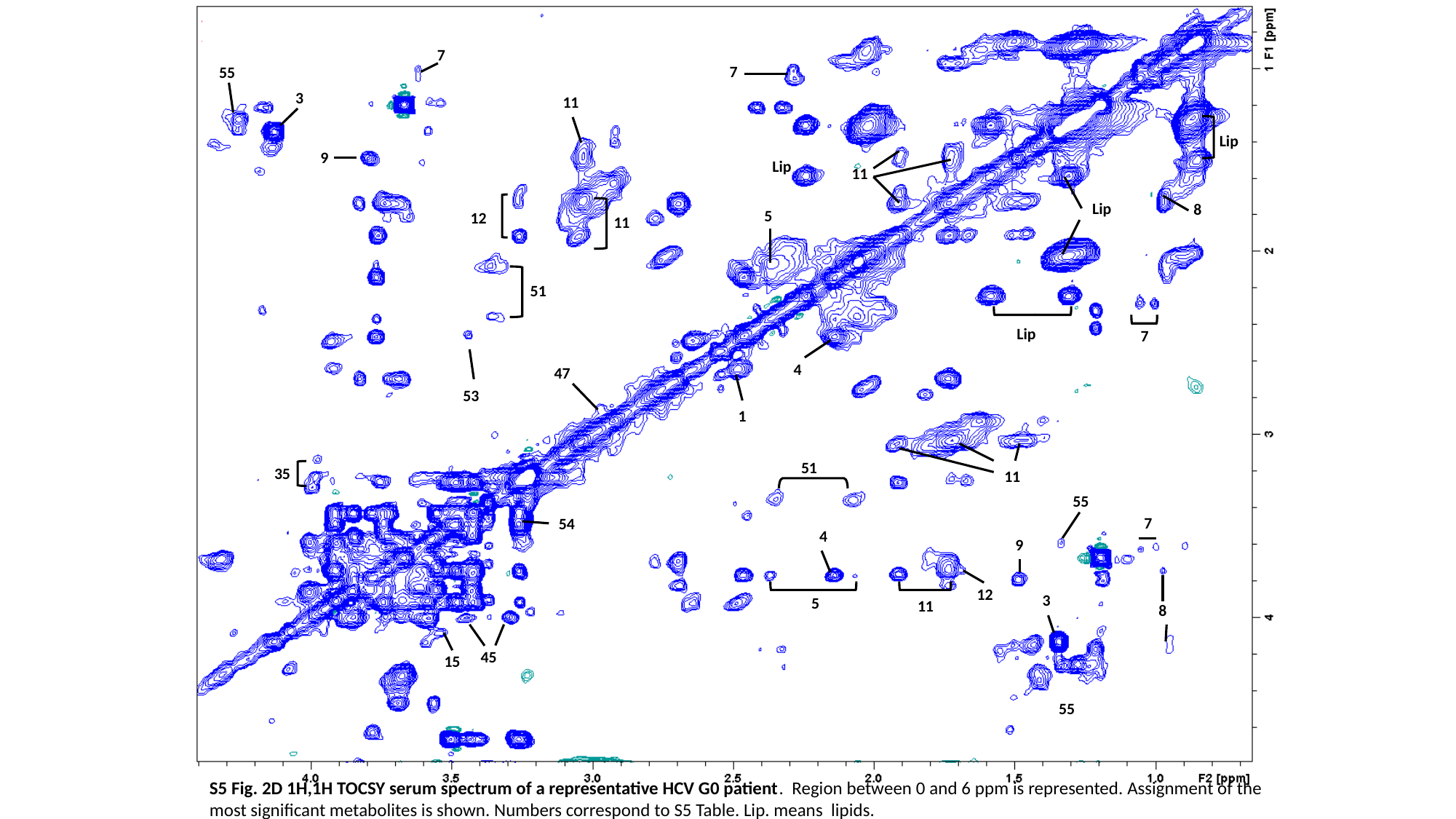

7
7
3
11
Lip
9
Lip
11
Lip
8
5
12
 11
51
Lip
7
 4
47
1
51
35
11
55
7
 4
 9
12
 3
5
11
 8
45
15
55
55
53
54
S5 Fig. 2D 1H,1H TOCSY serum spectrum of a representative HCV G0 patient. Region between 0 and 6 ppm is represented. Assignment of the most significant metabolites is shown. Numbers correspond to S5 Table. Lip. means lipids.
